# Smallpox Disarmed

**Published:** 1899-11

**Authors:** 


A NOTABLE PAPER.

SMALLPOX DISARMED.—The paper of Dr. Bibb, on Bi-Chloride of Mercury Baths in Smallpox, published in this issue, should attract wide attention and be extensively reproduced because of its immense importance and great practical value. If further observation should confirm his statements, and no doubt it will, Dr. Osborn's discovery deserves to rank second in importance to that of Jenner. Smallpox will be disarmed of its terrors and its dangers. So long as civilized governments will insist on "stamping out" smallpox rather than take steps to prevent and finally exterminate it, *i. e.*, by a system of compulsory vaccination, as insisted upon by the JOURNAL for years, it is well enough to know how to cure it and disinfect the patient at the same time. Bi-Chloride of mercury baths will become to smallpox what antitoxin is to diphtheria. Dr. Osborn published his discovery in the *Texas Sanitarium* in May, 1894. But his observation at that time had not been confirmed nor, so far as we know, had his remedy been tried.

Dr. Bibb's paper is the most important paper of the decade.

